# Supplementary material for: The Effect of Plastic-Related Compounds on Transcriptome-Wide Gene Expression on CYP2C19-Overexpressing HepG2 Cells
Source: Molecules. 2023 Aug 8;28(16):5952. doi: 10.3390/molecules28165952 (PMC10459118; doi:10.3390/molecules28165952)
Supplement: Supplementary file 1 [file molecules-28-05952-s001.zip › molecules-2531193-supplementary.pdf]

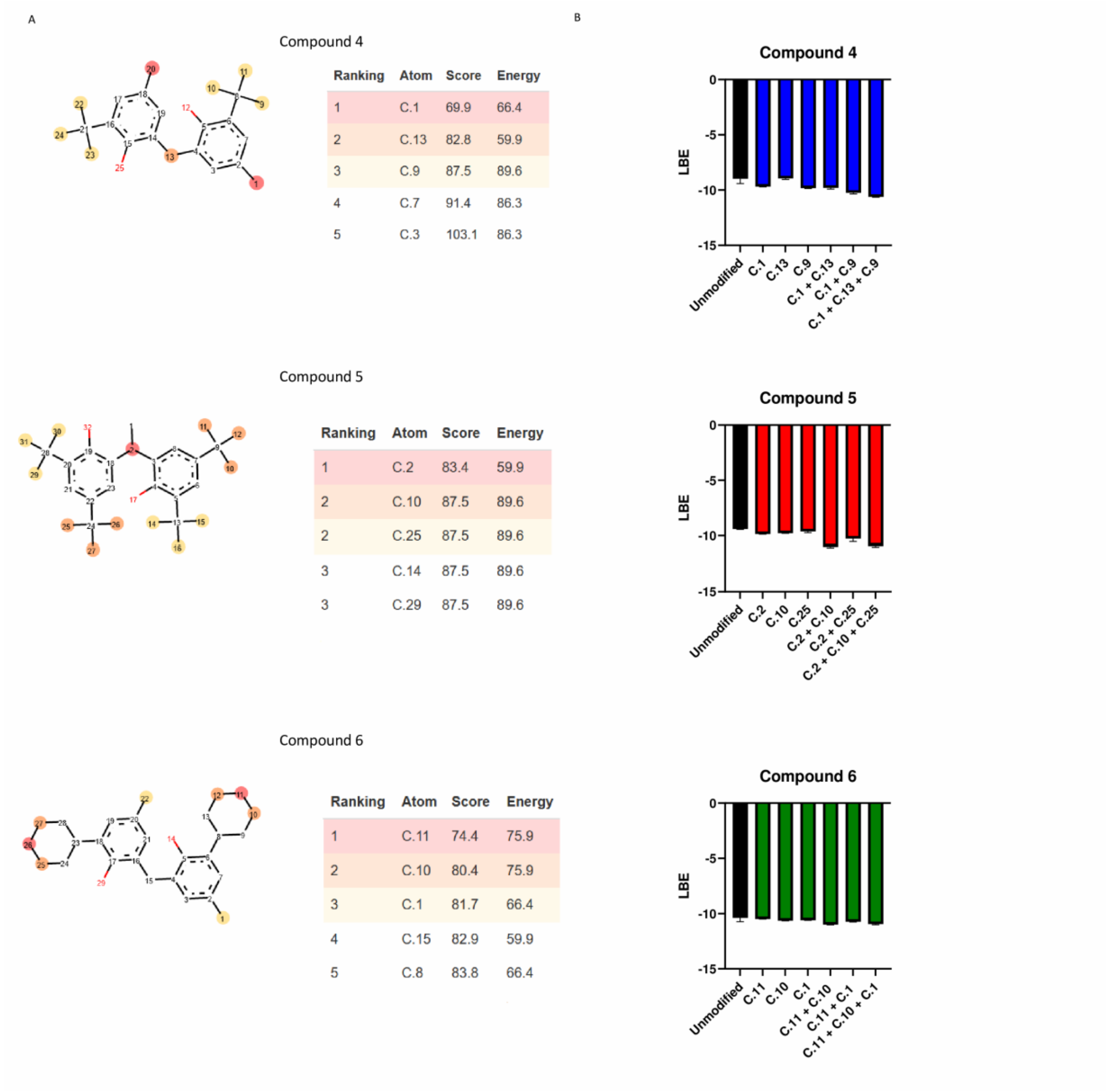

Compound 4

Ranking	Atom	Score	Energy
1	C.1	69.9	66.4
2	C.13	82.8	59.9
3	C.9	87.5	89.6
4	C.7	91.4	86.3
5	C.3	103.1	86.3

Compound 5

Ranking	Atom	Score	Energy
1	C.2	83.4	59.9
2	C.10	87.5	89.6
2	C.25	87.5	89.6
3	C.14	87.5	89.6
3	C.29	87.5	89.6

Compound 6

Ranking	Atom	Score	Energy
1	C.11	74.4	75.9
2	C.10	80.4	75.9
3	C.1	81.7	66.4
4	C.15	82.9	59.9
5	C.8	83.8	66.4

Supplementary Figure S1. Predicted sites of metabolism and lowest binding energy (LBE) for the selected compounds. (A) Possible sites of hydroxylation by CYP2C19 for the three selected compounds with the top 5 ranking positions. (B) Lowest binding energies (LBE) of the metabolites of the various compounds in different combinations in comparison with the unmodified molecule.
